# Advancing the pathologic phenotype of giant axonal neuropathy: early involvement of the ocular lens

**DOI:** 10.1186/s13023-018-0957-5

**Published:** 2019-02-01

**Authors:** Diane Armao, Thomas W. Bouldin, Rachel M. Bailey, Jody E. Hooper, Diana X. Bharucha, Steven J. Gray

**Affiliations:** 1 Department of Pathology and Laboratory Medicine, University of North Carolina School of Medicine, Chapel Hill, NC USA; 2 Department of Radiology, University of North Carolina School of Medicine, Chapel Hill, NC USA; 3 Gene Therapy Center, University of North Carolina at Chapel Hill Chapel Hill, Chapel Hill, NC USA; 4 Department of Pediatrics, University of Texas Southwestern Medical Center, Dallas, TX USA; 5 Department of Pathology, Johns Hopkins University, Baltimore, MD USA; 6 Department of Neurology and Pediatrics, Children’s National Health System, Washington, DC USA; 7 National Institutes of Health NINDS/ Neurogenetics Branch, Bethesda, MD USA; 8 Department of Ophthalmology, University of North Carolina School of Medicine, Chapel Hill, NC USA; 9 Department of Molecular Biology, University of Texas Southwestern Medical Center, Dallas, TX USA; 10 Department of Neurology and Neurotherapeutics, University of Texas Southwestern Medical Center, Dallas, TX USA

**Keywords:** Giant axonal neuropathy (GAN), Intermediate filaments (IF), GAN KO mouse model, Gigaxonin, Human GAN, Lens epithelium, IF accumulations

## Abstract

Giant axonal neuropathy (GAN; ORPHA: 643; OMIM# 256850) is a rare, hereditary, pediatric neurodegenerative disorder associated with intracellular accumulations of intermediate filaments (IFs). GAN knockout (KO) mouse models mirror the IF dysregulation and widespread nervous system pathology seen in human GAN. Validation of therapeutic efficacy and viral vector delivery systems with these GAN KO models has provided the springboard for the development of a viral vector being delivered intrathecally in an ongoing Phase I gene therapy clinical trial for the treatment of children with GAN (https://clinicaltrials.gov/ct2/show/NCT02362438). During the course of a comprehensive pathologic characterization of the GAN KO mouse, we discovered the very early and unexpected involvement of the ocular lens. Light microscopy revealed the presence of intracytoplasmic inclusion bodies within lens epithelial cells. The inclusion bodies showed strong immunohistochemical positivity for glial fibrillary acidic protein (GFAP). We confirmed that intracytoplasmic inclusion bodies are also present within lens epithelial cells in human GAN. These IF inclusion bodies in lens epithelial cells are unique to GAN. Similar IF inclusion bodies in lens epithelial cells have not been reported previously in experimental animal models or human diseases. Since current paradigms in drug discovery and drug repurposing for IF-associated disorders are often hindered by lack of validated targets, our findings suggest that lens epithelial cells in the GAN KO mouse may provide a potential target, in vivo and in vitro, for evaluating drug efficacy and alternative therapeutic approaches in promoting the clearance of IF inclusions in GAN and other diseases characterized by intracellular IF accumulations.

**Electronic supplementary material:**

The online version of this article (10.1186/s13023-018-0957-5) contains supplementary material, which is available to authorized users.

Giant axonal neuropathy (GAN, OMIM# 256850) is a rare, hereditary, pediatric neurodegenerative disorder associated with intracellular accumulations of intermediate filaments (IFs) [1]. The disease affects both the peripheral nervous system (PNS) and central nervous system (CNS), and patients nearly always succumb to disease by the third decade. The pathologic signature of GAN in the PNS and CNS is giant axonal swellings filled with dense accumulations of whorled, structurally normal neurofilaments. GAN is caused by autosomal recessive loss-of-function mutations in the GAN gene that encodes the protein gigaxonin. Gigaxonin plays a pivotal role in the cytoskeletal organization and degradation of IFs. Loss of gigaxonin leads to accumulation of different types of IFs within a variety of cells, including desmin in muscle cells, vimentin in fibroblasts, neurofilaments in neurons, and glial fibrillary acidic protein (GFAP) in astrocytes [2]. Most GAN patients also have characteristically tightly curled hair due to alterations of keratin IFs [3].

Three mouse models of GAN have been developed by knocking out part of the endogenous GAN gene [4–6]. All three mouse models mirror the IF dysregulation and widespread nervous system pathology seen in human GAN [7]. Validation of therapeutic efficacy and viral vector delivery systems with these GAN KO models [8] has provided the springboard for the development of a viral vector to be delivered intrathecally in a Phase I gene therapy clinical trial for the treatment of children with GAN [9].

During the course of a comprehensive study of the pathologic findings in the GAN KO mouse, we encountered the unexpected and very early involvement of the ocular lens (Fig. 1). Here, described for the first time, we document the early appearance of abundant IF accumulations in lens epithelial cells of the GAN KO mouse. Lens epithelial cells potentially provide an easily accessible target for accelerating complementary drug discovery and drug repurposing strategies for human GAN.

**Fig. 1:**
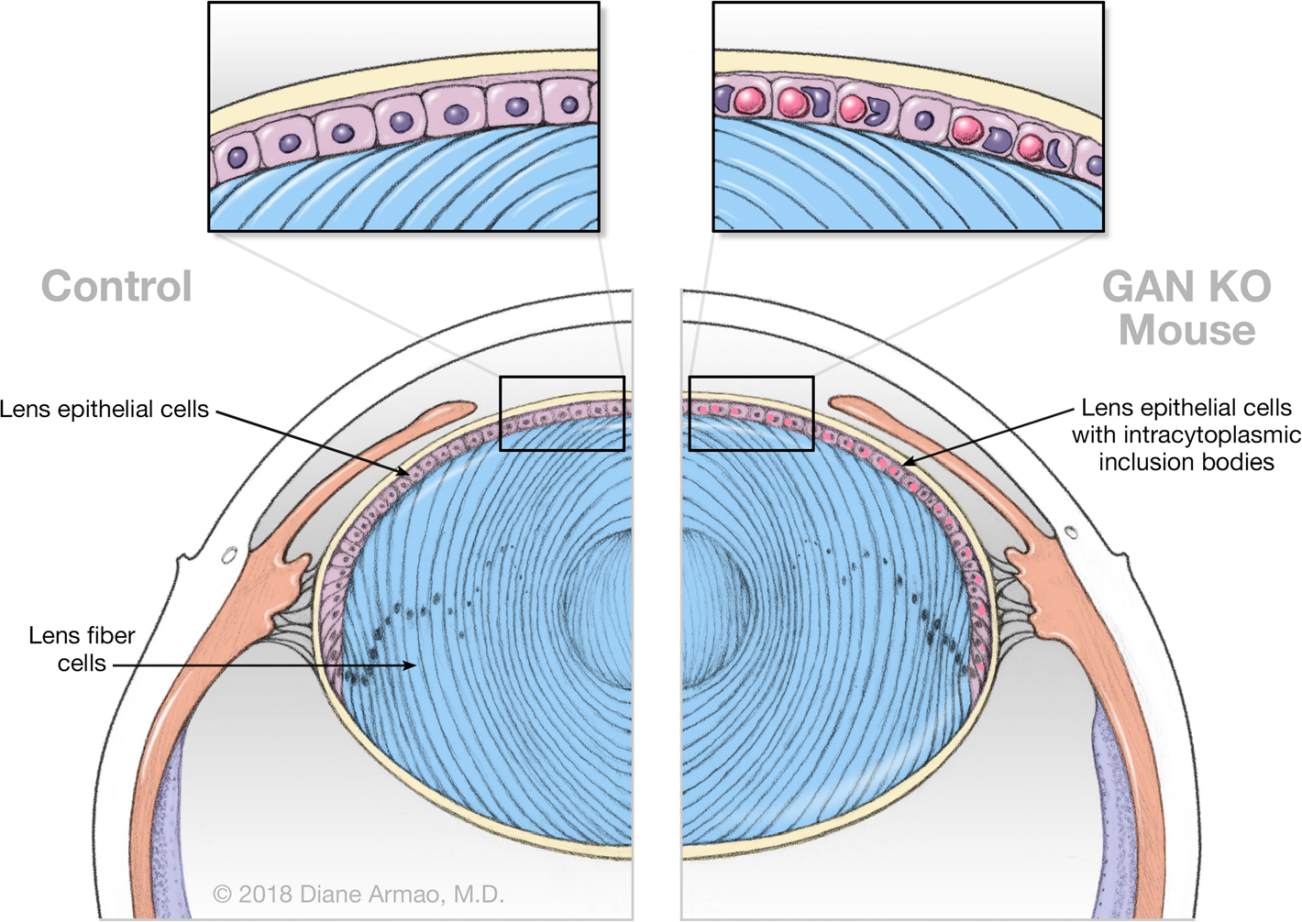
Ocular Lens. Control. Lens cells exist in two distinct forms, lens epithelial cells and lens fiber cells. The anterior surface of the lens is covered by a single layer of epithelial cells that serve as a reservoir for continual lens fiber cell formation and lens growth throughout life. The lens is unique as reflected in almost continuous cell production with negligible cell loss. On their path to becoming mature lens fiber cells, lens epithelial cells undergo extraordinary structural differentiation [10]. GAN KO mouse. Oval intracytoplasmic eosinophilic inclusion bodies within lens epithelial cells

GAN KO mice with a deletion of GAN exons 3–5 (GAN/Y) [4] or a deletion of GAN exon 1 (GAN/J) [6] were maintained at the University of North Carolina at Chapel Hill (UNC–CH) as previously described [8]. Heterozygous GAN mice are phenotypically normal [4, 6] and were used as controls. Mixed sex and age-matched littermates from both GAN KO models were used in these studies (4-month-old cohort: 4 KO, 2 heterozygotes; 24-month-old cohort: 10 KO, 15 heterozygotes).

In 4-month-old GAN KO mice, light microscopic examination of H&E-stained sections revealed oval, intracytoplasmic eosinophilic inclusion bodies within lens epithelial cells (Fig. 2a). Histologically identical inclusion bodies were found in 24-month-old GAN KO mice (Fig. 2b). In both 4-month-old and 24-month-old cohorts, inclusion bodies were present in almost every epithelial cell. A panel of immunohistochemical stains for lens IF proteins (GFAP, vimentin, keratin 8/18, CP49 and filensin) [10] showed strong immunoreactivity of inclusion bodies for GFAP (Fig. 2c). The epithelial cell inclusion bodies were present in both GAN/J and GAN/Y KO mice. Age-matched control mice had no inclusion bodies (Fig. 2d). The inclusion bodies were not present in lens fiber cells in the GAN KO mice or age-matched controls. No lens fiber cell degeneration was identified histologically in 4-month-old GAN KO mice or age-matched controls. Lens fiber cell degeneration, morphologically consistent with age-related degeneration [11], was present to a similar degree in both 24-month-old GAN KO mice and age-matched controls.

**Fig. 2:**
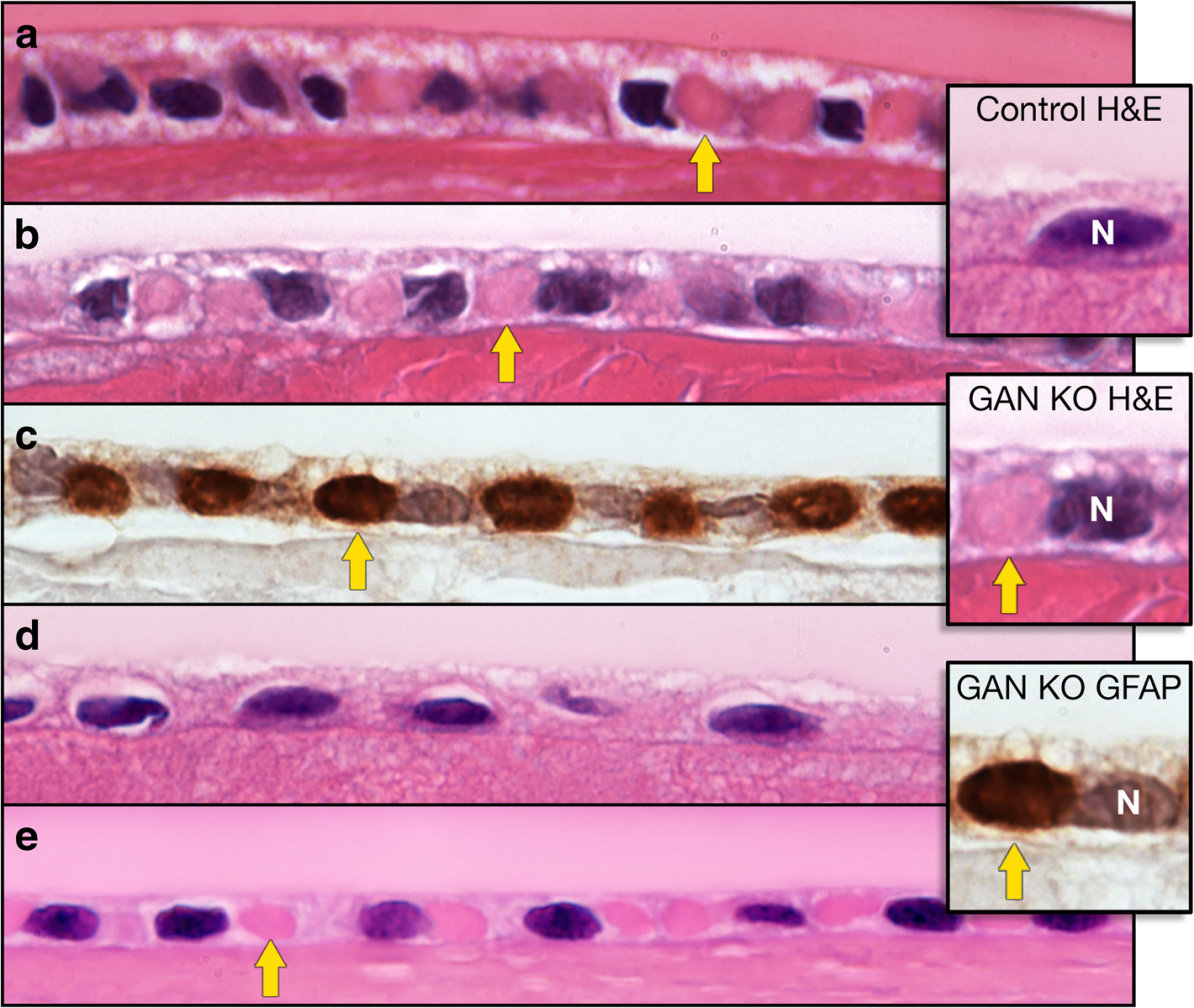
Lens epithelial cells in GAN KO mice, age-matched controls and human GAN. **a.** GAN KO (4-month-old) lens epithelial cells show intracytoplasmic inclusion bodies (H&E original magnification 100X). **b.** GAN/J KO (24-month-old) lens epithelial cell inclusion bodies (H&E original magnification 100X). **c.** GAN/J KO (24-month-old) lens epithelial cell inclusion bodies show strong GFAP immunoreactivity (GFAP IHC original magnification 100X). **d.** Control mouse (24-month-old) histologically unremarkable lens epithelial cells (H&E original magnification 100X). **e.** Human GAN lens epithelial cells reveal intracytoplasmic inclusion bodies (H&E original magnification 100X, formalin fixed, paraffin embedded tissue. Decedent was a young child with phenotypically typical GAN) (*arrows point to one of the numerous intracytoplasmic inclusion bodies*). Inset. Lens epithelial cells. Control mouse (24-month-old) (H&E); GAN/J KO (24 -month-old) lens epithelial cell inclusion body (H&E); GAN/J KO (24-month-old) lens epithelial cell inclusion body shows strong GFAP immunoreactivity (GFAP IHC). (***N*** *designates nucleus, arrow points to cytoplasmic inclusion body*)

The neuropathological phenotype of the GAN KO mouse model shares many morphological features with the human disease [7]. Here, described for the first time in the GAN KO mouse, we document the presence of intracytoplasmic IF inclusion bodies in lens epithelial cells. The inclusion bodies were present in the young 4-month-old KO mice and served as a reliable, easily identifiable, early marker of GAN.

These IF inclusion bodies in lens epithelial cells appear to be unique to GAN, as similar IF inclusion bodies have not been reported previously in experimental animal models or human diseases. Although lens abnormalities have not been reported in clinical or postmortem studies of human GAN [12–16], we confirmed in a specimen obtained at autopsy that similar appearing, intracytoplasmic inclusion bodies are also present in lens epithelial cells in human GAN (Fig. 2e).

The presence of GFAP-positive inclusion bodies in lens epithelial cells and their absence in lens fiber cells is intriguing. One difference between lens epithelial cells and lens fiber cells is the large concentration of the chaperone protein complex alpha-crystallin in lens fiber cells [17]. The chaperone activity of alpha-crystallin modulates the assembly of IFs, including GFAP, and assists IFs in recovery from stress by preventing inappropriate filament-filament interactions that would otherwise lead to aggregation [18].

Current paradigms in drug discovery and drug repurposing for IF-associated disorders are often hindered by lack of validated targets [19]. One strategy to circumvent this constraint is to screen against a disease phenotype in cell culture or animal model that recapitulates the pathologic phenotype of the human disease [19, 20]. Our findings suggest that lens epithelial cells in the GAN KO mouse may provide a potential target cell, in vivo, for evaluating the efficacy of drugs and other therapeutic approaches in promoting clearance of IF inclusions. Additionally, lens epithelial cells can be grown on their native basement membrane or as dissociated cells [21] and serve as a simple in vitro model system of target cells.

Intracytoplasmic accumulations of IFs are a distinctive pathological feature shared by common neurodegenerative diseases of adulthood, such as Alzheimer’s disease and Parkinson’s disease, as well as rare neurodegenerative diseases of childhood, such as Alexander disease and GAN [2]. It is possible that lens epithelial cells from the GAN KO mouse, if used as a drug repurposing screen, could be extended to address multiple diseases that share an IF accumulation pathologic phenotype [20, 22].

In summary, the GAN KO mouse exhibits great fidelity to the characteristic pathologic features and selected functional deficits of human GAN [7]. Here, we present the novel finding of GAN pathology in both mouse and human lens epithelial cells. We suggest that lens epithelium may serve as a target tissue in which to study the effects of pharmacological interventions on GAN and potentially other disorders characterized by intracytoplasmic IF accumulations.

## Additional files

Additional file 1: Details regarding experimental procedures and immunohistochemistry (IHC). (DOCX 15 kb)

Additional file 2: Details regarding immunoreactivity (IR). (DOCX 16 kb)

## Abbreviations

CNS: Central nervous system
GAN: Giant axonal neuropathy
GFAP: Glial fibrillary acidic protein
IF: Intermediate filament
KO: Knockout
PNS: Peripheral nervous system
