# Supplementary material for: Advancing the pathologic phenotype of giant axonal neuropathy: early involvement of the ocular lens
Source: Orphanet J Rare Dis. 2019 Feb 1;14:27. doi: 10.1186/s13023-018-0957-5 (PMC6359799; doi:10.1186/s13023-018-0957-5)
Supplement: Supplementary file 1 — Details regarding experimental procedures and immunohistochemistry (IHC). (DOCX 15 kb) [file 13023_2018_957_MOESM1_ESM.docx]

Additional file 1: Supplementary Information-Methods. Details regarding experimental procedures and immunohistochemistry (IHC).

Procedures

Mixed sex and age-matched littermates from both GAN KO models were used in these studies (4-month-old cohort: 4 KO, 2 heterozygotes; 24-month-old cohort: 10 KO, 15 heterozygotes). Mice were anesthetized with an overdose of avertin (0.04 mL/g of a 1.25% solution) and then perfused with phosphate-buffered saline containing 1 µg/mL heparin. For the 4-month-old cohort, eyes were immediately dissected and immersion-fixed in 10% neutral buffered formalin (NBF). For the 24-month-old cohorts, mice were additionally perfused with 4% paraformaldehyde before eyes were removed and fixed in 10% NBF. For a subset of animals analyzed with CP49 and filensin antibodies eyes were fixed as previously described (1). Briefly, eyes were removed and drop-fixed in -80^o^C methanaol:acetic acid (97:3) and stored at -80^o^C for 48 hours. Tubes were moved to -20^o^C for 4 hours and to room temperature for 2 hours. Methanol-acetic acid was then exchanged for 100% ethanol. Intact globes were parafﬁn embedded and cross-sectioned at 5-µm thickness using a microtome.

Immunohistochemistry

Serial sections were stained with hematoxylin and eosin (H&E) and with a panel of antibodies against lens IF proteins (GFAP, vimentin, keratin 8/18, CP49 and filensin) (2) and were counterstained with hematoxylin. Slides were deparafinnized using standard methods and antigen retrieval was performed with antigen unmasking solution (Vector H-3300) by incubating tissues at 60^o^C overnight in a water bath. Antibodies used were rabbit monoclonal anti-vimentin (Abcam ab92547; 1:800), rabbit polyclonal anti-GFAP (Dako Z0334; 1:2000), rabbit monoclonal anti-cytokeratin 8/18 (Biocare Medical API31161 AA; ready-to-use-concentration), and rabbit polyclonals anti-CP49 and anti-filensin (generously provided by Dr. Paul FitzGerald, University of California Davis; 1:500). The secondary antibody used was biotinylated anti-rabbit (Vector BA-1000; 1:200). Images were captured at 100X with oil immersion using the Olympus BX61 microscope (Olympus America) and the QImaging Retiga 4000R camera (Surrey). Slides were reviewed by two microscopists that were blinded to genotype.

**References**

1. Sun W, Zheng W, Simeonov A. Drug discovery and development for rare genetic disorders. Am J Med Genet A. 2017; 173(9):2307-22.

2. Song S, Landsbury A, Dahm R, Liu Y, Zhang Q, Quinlan RA. Functions of the intermediate filament cytoskeleton in the eye lens. J Clin Invest. 2009;119(7):1837-48.
