# Supplementary material for: Advancing the pathologic phenotype of giant axonal neuropathy: early involvement of the ocular lens
Source: Orphanet J Rare Dis. 2019 Feb 1;14:27. doi: 10.1186/s13023-018-0957-5 (PMC6359799; doi:10.1186/s13023-018-0957-5)
Supplement: Supplementary file 2 — Details regarding immunoreactivity (IR). (DOCX 16 kb) [file 13023_2018_957_MOESM2_ESM.docx]

Additional file 2: Supplementary Information-Results. Details regarding immunoreactivity (IR).

In GAN KO mice lens epithelial cells, the cytoplasm surrounding the inclusion bodies showed immunoreactivity (IR) for vimentin. Lens epithelial cell inclusion bodies showed no IR for vimentin. Lens epithelial cell cytoplasm and inclusion bodies showed no IR for K8/K18. Conventional immunohistochemistry for filensin and CP49 on GAN KO mice showed the expected IR in the outer cortical fiber cells (1) but no IR in the inclusion bodies. The epithelial cell inclusion bodies were present in both GAN/J and GAN/Y mice. Age-matched controls showed expected cytoplasmic IR for vimentin and GFAP, as described in both mouse and human lens epithelial cells (2, 3).

**References**

1. FitzGerald PG. Lens intermediate filaments. Exp Eye Res. 2009;88(2):165-72.

2. Song S, Landsbury A, Dahm R, Liu Y, Zhang Q, Quinlan RA. Functions of the intermediate filament cytoskeleton in the eye lens. J Clin Invest. 2009;119(7):1837-48.

3. Bozanic D, Bocina I, Saraga-Babic M. Involvement of cytoskeletal proteins and growth factor receptors during development of the human eye. Anat Embryol (Berl). 2006;211(5):367-77.
